# Lifetime Clinical Course of Hypertrophic Cardiomyopathy: Outcome of the Historical Florence Cohort Over 5 Decades

**DOI:** 10.1016/j.jacadv.2023.100337

**Published:** 2023-05-24

**Authors:** Niccolò Maurizi, Iacopo Olivotto, Martin S. Maron, Giacomo Bonacchi, Panagiotis Antiochos, Benedetta Tomberli, Carlo Fumagalli, Corrado Poggesi, Martina Berteotti, Francesca Girolami, Franco Cecchi, Barry J. Maron

**Affiliations:** a Cardiomyopathy Unit, Careggi University Hospital, Florence, Italy; b Service of Cardiology, University Hospital of Lausanne, Lausanne, Switzerland; c Department of Experimental and Clinical Medicine, University of Florence, Florence, Italy; d Hypertrophic Cardiomyopathy Center, Lahey Hospital and Medical Center, Burlington, Massachusetts, USA; e Cardiology Unit, Meyer University Hospital, Florence, Italy; f Department of Cardiovascular, Neural and Metabolic Sciences, Center for Cardiac Arrhythmias of Genetic Origin and Laboratory of Cardiovascular Genetics, IRCCS Istituto Auxologico Italiano, San Luca Hospital, Milan, Italy

**Keywords:** heart failure, hypertrophic cardiomyopathy, long-term outcome, sudden cardiac death

## Abstract

**Background:**

The current understanding of the clinical course and long-term outcome of patients with hypertrophic cardiomyopathy (HCM) has been extrapolated from cohorts with relatively short follow-up, usually <10 years. Extended assessments more closely reflecting HCM lifetime burden are not available.

**Objectives:**

The purpose of this study was to report the lifetime clinical course of HCM.

**Methods:**

We analyzed the clinical course of HCM patients diagnosed at our center from 1970 to 1992 and followed annually to the present. Cumulative incidence functions were used to estimate the incidence of HCM-related mortality (including heart failure [HF]/stroke related, sudden cardiac death [SCD]) and non-HCM related.

**Results:**

A total of 202 patients (age 41 ± 17 years; 63% male) were followed 27 ± 6 [range: 3-50] years. Overall, 97 (48%) survived and 105 (52%) died during the particularly long follow-up; 69 deaths were related to HCM, including 53 HF related, 11 fatal embolic strokes, and 16 SCDs. Annual overall HCM-related mortality was 1.3%/y, increasing from 0.7% during the first decade to 1.8% in the second/third decade (*P* < 0.01), mainly driven by increase in HF-/stroke-related events (from 0.6% to 1.3%). The SCD mortality rate was similar in the 2 periods (0.1% vs 0.44%, *P* = 0.10). Of the 69 HCM deaths, 29 (42%) occurred before the widespread availability of effective contemporary treatment strategies and are considered potentially preventable.

**Conclusions:**

In this unique HCM cohort followed for up to 50 years, often before contemporary therapies became widely implemented for HCM, HF frequently progressed over time, while arrhythmic SCD events were less common and remained constant over time. Despite spanning different management eras over 5 decades, HCM-related mortality remained relatively low (1.3%/y).

HCM is the most common inherited cardiomyopathy, characterized by a heterogeneous phenotype and clinical course.^1^^,^^2^ Current understanding of the clinical course, long-term burden, and longevity of HCM patients has been extrapolated from cohorts with follow-up <10 years,^3^, ^4^, ^5^, ^6^, ^7^, ^8^, ^9^, ^10^, ^11^, ^12^ potentially underestimating the lifetime burden of the disease^13^ and impacting our appreciation for risk stratification and therapy. To date, observations spanning longer periods of follow-up are lacking in the literature. Therefore, the objective of this study was to examine the clinical course and cardiac outcomes of a large historic HCM cohort, originally reported in 1995^13^ and followed longitudinally up to 50 years.

## Methods

### Study population

Data pertaining to the clinical status and management of a previously assembled cohort of 202 patients with hypertrophic cardiomyopathy (HCM) seen at our center between 1970 and 1992^14^ were reviewed. The diagnosis of HCM was based on 2-dimensional echocardiographic evidence of a hypertrophied, nondilated LV (maximum wall thickness ≥15 mm, or the equivalent relative to body surface area in children), in the absence of another cardiac or systemic disease capable of producing the magnitude of hypertrophy evident. The peak left ventricular (LV) outflow tract (LVOT) velocity was averaged from 3 to 5 cardiac cycles recorded at a sweep speed of 50 to 100 mm/s. Outflow obstruction was defined by Doppler echocardiography as a systolic anterior motion of the mitral valve and LVOT (subaortic) velocity of >2.7 m/s at rest or >3.5 m/s with provocation, which is respectively comparable to an outflow gradient >30 mm Hg at rest or >50 mm Hg with provocation. In all cases, particular consideration was given to distinguishing the Doppler signal of LVOT obstruction from that of mitral regurgitation.

Patients were followed annually or more often if necessary by clinical evaluation including noninvasive testing on average 27 years (ranging to 50 years). Follow-up was defined as the duration from study entry to last visit (or death), by clinical visit or telephone, regularly updated to 2020 (9 patients were lost to the 2020 follow-up). All study patients came from Tuscany or in the adjacent region of Umbria. HCM phenocopies (Fabry disease, amyloidosis, and LAMP2 [Danon] cardiomyopathy) were excluded. Diagnosis and management throughout the entire period was the responsibility of 2 senior cardiologists (F.C. and I.O.). The study was approved by the institutional review board; informed consent was waived, this being an anonymized retrospective study. The data that support the findings of this study are available from the corresponding author upon reasonable request.

### Study end points

Overall mortality was divided into HCM-related and non–HCM-related causes. HCM-related deaths included heart failure (HF-)/stroke-related deaths or sudden cardiac deaths (SCDs). HF-/stroke-related deaths were defined as events occurring in the context of cardiac decompensation and progressive disease course, including when complicated by pulmonary edema or evolution to the end-stage HF, or due to fatal embolic stroke in the context of atrial fibrillation (AF). SCD was defined as sudden and unexpected collapse occurring <1 hour from the onset of symptoms in patients previously experiencing relatively stable or uneventful clinical course. Potentially lethal cardiovascular events in which patients were either successfully resuscitated from cardiac arrest or received appropriate defibrillation therapy from an implantable cardioverter-defibrillator (ICD) were not regarded as equivalent to sudden death. Likewise, patients with advanced refractory HF who received heart transplantation were not considered surrogates of HF-/stroke-related deaths.

### Impact of evolving treatment strategies on outcome

The present study analyzes the long-term follow-up of this historical cohort, dating back to the period only shortly after the initial pathologic description of HCM in 1960 by Teare and the first comprehensive clinical description by Braunwald.^15^ Thus, overall clinical outcome reflects the interplay of the natural history of the disease and evolving management strategies, since certain current advanced treatment options were not widely available in Central Italy for portions of the extended follow-up period. For example, ICDs for primary prevention of SCD were not employed until about 2000 or later for HCM.^2^^,^^16^ Access to surgeons experienced in the septal myectomy operation has been inconsistent in Italy through the years, and with the cultural obstacle in which patients have often been reluctant to travel great distances or internationally for operations and therefore were largely managed by reliance on negative inotropic drugs to reduce LV outflow obstruction. A surgical myectomy program was first available in Florence since 2005, while alcohol septal ablations have been performed since 2000. In addition, in the early years of the cohort, routine anticoagulation was inconsistently prescribed in accord with evolving indications for HCM patients, and there was limited access to heart transplant programs.

Thus, to assess the potential impact of inconsistent access to certain contemporary treatments in the early phases of our follow-up, the proportion of HCM-related deaths that were potentially preventable by contemporary management strategies was calculated. Such events included: 1) fatal embolic strokes occurring in patients with known AF who were not taking oral anticoagulants; 2) patients who died suddenly without an ICD in the presence of ≥1 established SCD risk factors^17^; 3) patients in NYHA functional classes III-IV with LV outflow gradients >50 mm Hg eligible for invasive septal reduction therapy but who were treated conservatively for HF; and 4) patients aged <65 years old who were candidates for heart transplant but died of HF.

### Statistical analyses

Comparisons of continuous variables, reported as mean ± SD or as median (IQR) for nonnormal distributions, were performed using paired Student’s *t*-test or nonparametric tests, as appropriate. Categorical variables, reported as counts and percentages, were compared between groups with paired chi-square or Fisher exact tests. Cumulative incidence function curves were employed to avoid bias introduced by competing risks to estimate the incidence of all-cause mortality and cause-specific mortality, including HCM-related death, HF/stroke death, and SCD death at follow-up in the study cohort. Patients lost to follow-up (n = 9) were censored at the last clinical contact.

Moreover, prediction of the event rate in the setting of competing risks was calculated by a Fine-Gray regression model, which allows estimation of the effect of covariates on the cumulative incidence function accounting for the effects of competing risks from non–HCM-related deaths.^18^ A 2-sided value of *P* < 0.05 was considered statistically significant. All analyses were performed using SPSS Statistics for Macintosh version 25.0 (IBM) and SAS version 9.4 (SAS Institute).

## Results

### Clinical and echocardiographic profile

The 202 consecutive HCM study patients were followed for 27 ± 6 years (range 3-50 years), from a mean age of 41 ± 17 years at enrollment to 68 ± 14 years at the most recent evaluation or death, with 49/202 (24%) surviving to >70 years. At baseline, 183 patients were asymptomatic or had mild symptoms (90%, in NYHA functional class I-II), 40 had LV outflow obstruction >30 mm Hg at rest (20%), and 21(10%) had AF (Table 1).

**Table 1:** Clinical Characteristics of the 202 HCM Patients at Baseline and Survivors at Each Follow-up

	Enrollment (n = 202)	First Follow-Up Analysis Survivors (n = 189)	Second Follow-Up Analysis Survivors (n = 97)
Medical history			
Age (y)	41 ± 17	52 ± 16	68 ± 14
Male	140 (69%)	133 (65%)	58 (60%)
NYHA functional class I-II	183 (90%)	154 (81%)	78 (80%)
NYHA functional class III-IV	19 (10%)	46 (25%)	19 (20%)
LVOT gradient >30 mm Hg (n)	40 (20%)	46 (24%)	23 (23%)
LVOT gradient ≥50 mm Hg (n)	23 (11%)	29 (15%)	9 (10%)
Syncope	8 (4%)	33 (17%)^a^	17 (18%)
Non sustained VT	22 (11%)	48 (25%)^a^	21 (22%)
Septal reduction interventions	0	5 (1%)	6 (5%)
Paroxysmal atrial fibrillation	10 (5%)	22 (12%)^a^	8 (8%)
Permanent atrial fibrillation	11 (5%)	35 (18%)	19 (20%)
ICD implantation	0	0	16 (16%)^a^
Pathogenic/likely pathogenic sarcomere variant	-	-	39/74 (52%)
Drugs			
Beta-blockers	18 (9%)	102 (54%)^a^	81 (83%)^a^
Verapamil	4 (2%)	6 (3%)	8 (8%)
Disopyramide	0	11 (6%)	14 (14%)
Amiodarone	8 (4%)	63 (33%)^a^	18 (19%)
ACE inhibitors	1 (0.1%)	7 (3%)	11 (11%)
Diuretics	31 (15%)	38 (20%)	35 (36%)
Anticoagulation	17 (8%)	28 (15%)	29 (30%)^a^
Echocardiography			
LA diameter (mm)	40 ± 6	41 ± 9	46 ± 5^a^
EF (%)	65 ± 9	64 ± 9	57 ± 11^a^
Maximal LV thickness (mm)	23 ± 5	24 ± 6	20 ± 3^a^
LV thickness ≥30 mm	24 (11%)	28 (15%)	10 (12%)
Restrictive diastolic pattern	12 (6%)	32 (17%)	22 (23%)^a^

Values are mean ± SD or n (%).

ACE = angiotensin-converting enzyme; ECG = electrocardiogram; EF = ejection fraction; HCM = hypertrophic cardiomyopathy; ICD = implantable cardioverter-defibrillator; LA = left atrium; LV = left ventricle; LVOT = left ventricular outflow tract; VT = ventricular tachycardia on ambulatory ECG.

a *P* < 0.05 to 0.001.

Progressive symptoms (to NYHA functional class III/IV) were significantly more common at most recent evaluation compared to initial visit (27% vs 10%, *P* < 0.01) (Supplemental Table 1). Syncope occurred in 54 (29%) and 83 (44%) had paroxysmal or permanent AF. Most patients were taking beta-blockers (n = 146, 85%) and 91 (48%) were on oral anticoagulants at most recent evaluation (Supplemental Table 1).

During follow-up, there was echocardiographic evidence of cardiac remodeling, including left atrial enlargement (increase in diameter, 40 ± 6 mm to 50 ± 9 mm; *P* < 0.01), reduction in maximal LV wall thickness (23 ± 5 mm to 20 ± 3 mm; *P* < 0.01), and decrease in LV ejection fraction (65% ± 9% to 54% ± 12%; *P* < 0.01; Supplemental Table 1). Of the 125 probands and relatives with genetic testing, 61 (48%) had a pathogenic/likely pathogenic mutation. Specifically, 37 (59%) of patients carried a variant in MYBPC3, MYH7 (13 [21%]), TNNT2 (6 [10%]), and TNNI3 (3 [4%]), while 2 others (4%) had double mutations in MYBPC3 and MYH7.

### Long-term clinical course and outcome

**Figure 1:**
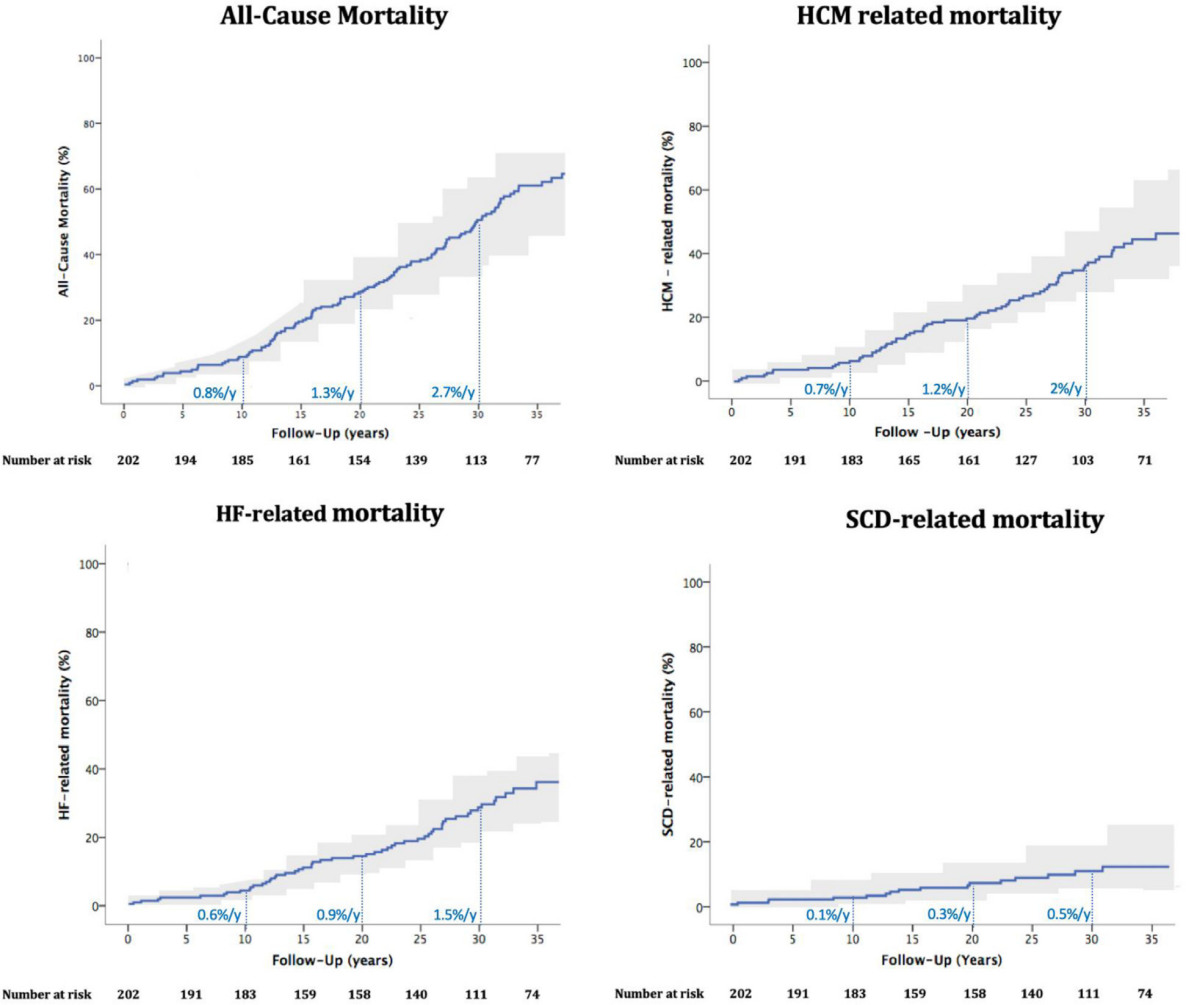
**Lifetime Clinical Course and Causes of Mortality in a HCM Cohort With Extended Follow-Up** Cumulative incidence function analysis of all-cause mortality **(top left)**, HCM-related deaths **(top right)**, heart failure-related deaths **(bottom left)**, and sudden cardiac death events **(bottom right)**. HCM = hypertrophic cardiomyopathy; HF = heart failure; SCD = sudden cardiac death.

During 27 ± 6 years of observation (5,153 person-years), 48% of the patients (97/202) had survived, including 49 (25%) >70 years and 52% of our patients have died (105/202) (Central Illustration). All-cause mortality was lower in the first decade of follow-up 0.8%/y (0.81 event per 100 person-years, 95% CI: 0.7-0.9), but increased in the second and third decade (1.3%/y [1.35 event per 100 person-years, 95% CI: 1.28-1.44] and 2.7%/y [2.74 event per 100 person-years, 95% CI: 2.65-2.90], respectively) (Figure 1). HCM was the primary cause of death in 69 patients, distinguished as 42 due to HF, 11 due to embolic stroke associated to AF, and 16 due to SCD (Central Illustration). The mean age at HCM death was 64 ± 11 years and 75% of death occurred after the age 55 (Supplemental Figure 1). The remaining 36 patients died (age at death 78 ± 9 years) due to cancer (n = 12), chronic obstructive airways disease (n = 7), complication of fractures in the elderly (n = 4), endocarditis (n = 3), end-stage renal disease (n = 3), abdominal aortic rupture (n = 1), myocardial infarction (n = 1), and multiple comorbidities not associated with HCM (n = 4).

**Central Illustration:**
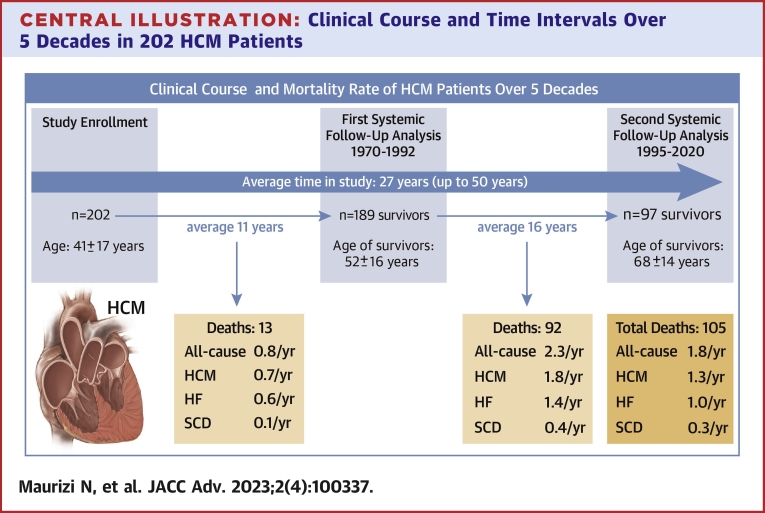
**Clinical Course and Time Intervals Over 5 Decades in 202 HCM Patients** CA = cardiac arrest; HCM = hypertrophic cardiomyopathy; HF = heart failure; SCD = sudden cardiac death

Mortality rate directly due to HCM was 1.3%/y (1.33 event per 100 person-years, 95% CI: 1.2-1.4), that is, 1.0% from HF-/stroke-related death (1 event per 100 person-years, 95% CI: 0.9-1.1) and 0.3% from SCD (0.3 event per 100 person-years, 95% CI: 0.2-0.4) (Figure 1). Patients with HF-/stroke-related deaths were older than those with SCD (69 ± 16 years vs 50 ± 18 years; *P* = 0.02) (Figure 1). The 30-year cumulative incidence of HCM death was 36.7% (95% CI: 33.4%-38.2%). Incidence rate of death directly due to HCM was 13.3 (95% CI: 12.8-13.6) cases per 1,000 person-years, 8.1 (95% CI: 7.9-8.4) cases per 1,000 person-years for HF-/stroke-related death, and 3.1 (95% CI: 3-3.5) per 1,000 person-years for SCD-related death.

In the first decade of follow-up, 13 HCM deaths occurred (11 HF/stroke related and 2 SCDs) with an annual mortality of 0.7% (95% CI: 0.6-0.9), of which 0.6% (95% CI: 0.5-0.7) from HF-/stroke-related complications and 0.1% (95% CI: 0.07-0.15) from SCD.^13^ In the second/third decade of observation, 56 HCM-related deaths occurred (42 HF/stroke related and 14 SCDs) with an annual mortality of 1.8% (95% CI: 1.7-1.9), of which 1.3% (95% CI: 1.2-1.4) from HF-/stroke-related complications and 0.4% (95% CI: 0.3-0.5) from SCD (Figure 1). The only independent predictor of HCM-related mortality over extended follow-up was a left atrial diameter >45 mm at diagnosis (HR: 1.3; 95% CI: 1.1-2.6; *P* = 0.04) (Table 2). Age at death of the age-matched Italian population from the same geographic region was 82 ± 12 (IQR: 74-86) years (Supplemental Figure 2).

**Table 2:** Regression Model Assessing the Relationship Between Clinical Risk Factors and HCM-Related Mortality

	Univariable Analysis			Multivariable Analysis		
	HR	95% CI	*P* Value	HR	95% CI	*P* Value
Male	0.8	0.5-3.6	0.1			
NSVT	1.2	0.9-4.3	<0.01			
Left atrial diameter >45 mm	5.4	3.1-10.2	<0.01	1.3	1.1-2.6	0.04
LVOT gradient >30 mm Hg	1.7	1.3-1.9	<0.01			
Unexplained syncope	1.9	2.3-11.1	<0.01	0.86	0.5-1.7	0.21
LV thickness ≥30 mm	2.3	1.8-2.9	<0.01	0.8	0.5-6.2	0.13
LV thickness regression (y)	1.5	1.1-8.2	<0.01			
Reduction in LV EF	2.8	1.4-4.1	<0.01			
CAD (y)	0.4	0.1-1.2	0.76			
Cancer (y)	0.2	0.1-0.9	0.88			
COPD (y)	0.5	0.5-1.1	0.32			
CKD (y)	0.8	0.5-2.3	0.65			

CAD = coronary artery disease; CKD = chronic kidney disease; COPD = chronic obstructive pulmonary disease; EF = ejection fraction; HCM = hypertrophic cardiomyopathy; LV = left ventricle; LVOT = left ventricular outflow tract; NSVT = nonsustained ventricular tachycardia.

Overall, 32 patients (17%) received an ICD, including 28 for primary and 4 for secondary prevention. Eleven of these 32 patients (33%) received appropriate ICD interventions terminating ventricular tachycardia/ventricular fibrillation, 1 to 11 years postimplant at age 27 ± 6 years. Three (1.5%) patients received inappropriate shocks (2 T-waves oversensing and 1 lead fracture), 1 needed lead-revision, and 1 was implanted with an S-ICD system because of transvenous lead failure. Fourteen patients underwent invasive septal reduction strategies without perioperative mortality (9 surgical septal myectomy and 5 alcohol septal ablation), aged 44 ± 15 years, with a preoperative LV gradient of 60 ± 29 mm Hg reduced to 15 ± 13 mm Hg postintervention. Four patients were transplanted at 51 ± 12 years (range 48-62 years), due to end-stage disease with systolic dysfunction (EF <50%), including one patient each with failed septal reduction intervention or with uncontrollable ventricular tachyarrhythmias. One of these 4 patients died from transplant-related complications at age 73 (11 years post-transplant).

Of the 42 patients who died of HF/stroke, 21 had obstructive disease and 21 were nonobstructive. The latter, evolved to end-stage with systolic dysfunction, and were managed medically, given the unavailability, ineligibility or rejection of a heart transplant. Patients with end-stage disease died at 69 ± 3 years, and compared to the non–end-stage group, were more frequently in permanent AF (17/42, 40% vs 24/160, 16%, *P* < 0.01) and more often had a pathogenic/likely pathogenic sarcomeric mutation (22/42, 51% vs 39/160, 24%, *P* < 0.01). Evidence of advanced cardiac remodeling was greater enlargement of left atrium (51 ± 5 mm vs 42 ± 7 mm, *P* < 0.01), reduced LV thickness (19 ± 5 mm vs 22 ± 6 mm, *P* < 0.01), and lower ejection fraction (44 ± 6 mm vs 59 ± 6 mm, *P* < 0.01), consistent with systolic dysfunction.

### Potential impact of contemporary treatment options

**Figure 2:**
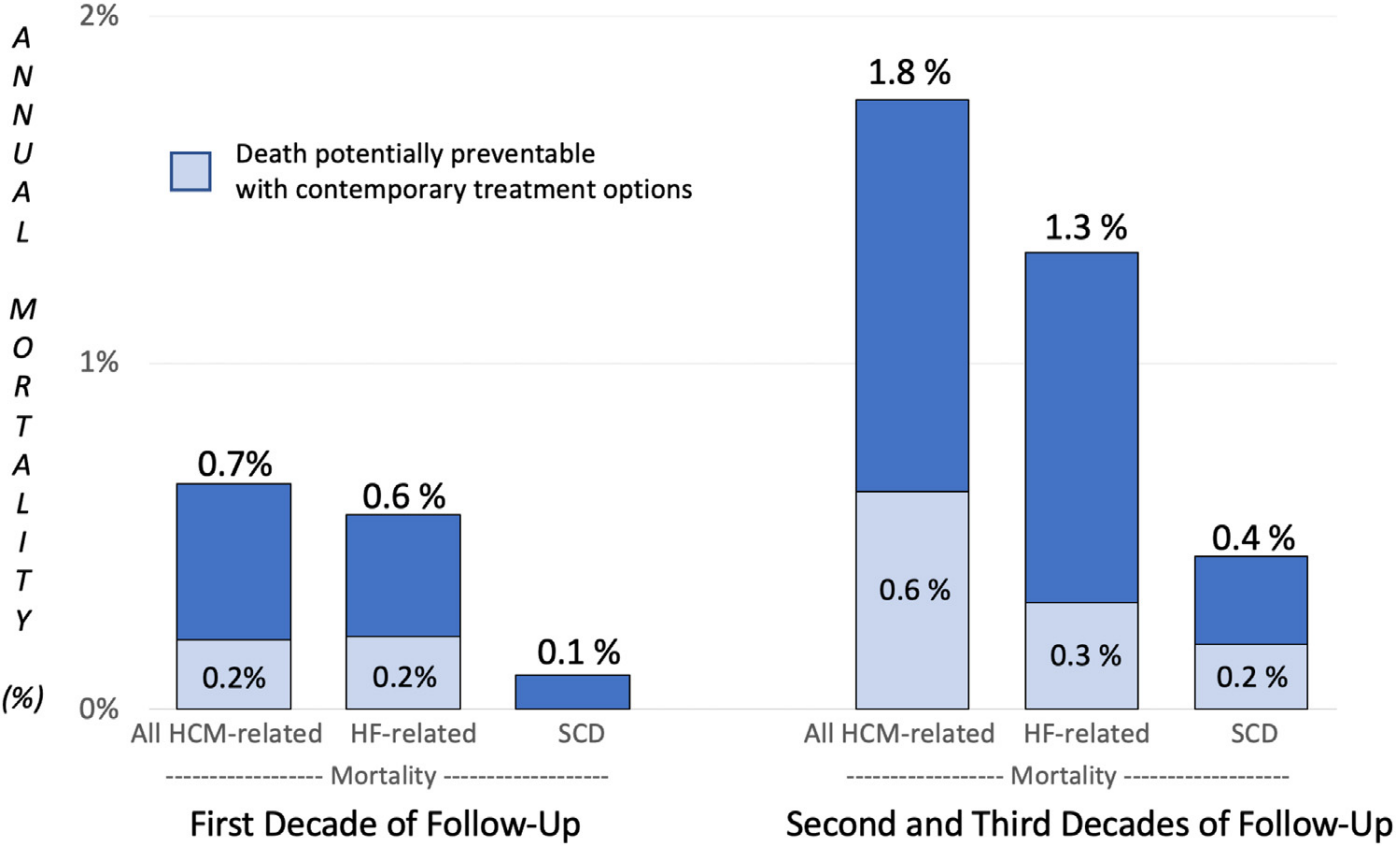
**Potential Impact of Contemporary Management Strategies on Risk for HCM Mortality** Estimated impact of current management approaches on HCM mortality due to heart failure or sudden deaths events, demonstrating the proportion of patients who would have benefited from treatment options not fully available to us 20 to 30 years ago. HCM = hypertrophic cardiomyopathy; HF = heart failure; SCD = sudden cardiac death.

Of the 69 deaths attributable to HCM, 29 (42%) occurred in earlier time period when currently available and effective treatment strategies may not have been accessible. These 29 patients, including 21 (aged 51 ± 16 years; range 21-71 years) with LV outflow gradient >50 mm Hg at rest/exercise and NYHA functional class III/IV symptoms who were treated conservatively without surgery and subsequently died in drug-refractory HF (5 in first decade and 16 during second/third decades); 2 nonobstructive patients aged <65 years, eligible for but not amenable clinically to heart transplant, who died in refractory HF; 2 patients with AF not compliant with oral anticoagulants who experienced fatal cardioembolic stroke; and 4 patients with ≥1 HCM SCD risk factors died suddenly without protection of a primary prevention ICD (including one who refused implant). Lack of availability of current contemporary treatment options early on for this cohort largely accounts for the higher mortality rate in the second/third decade of follow-up (Figure 2).

## Discussion

To our knowledge, the current study is the longest observational HCM cohort study, with follow-up averaging 27 years and ranging to 50 years. This unique assessment of lifetime HCM-associated outcomes demonstrated that HCM-related mortality was very low during the first decade (0.7%/y, as reported in 1995),^14^ but increased 2.5-fold, to 1.8%/y, in the second and third decades of follow-up, independent of age at diagnosis. This specific outcome was driven largely by a time-related increase in HF morbidity and mortality, ultimately causing death in about 25% of the initial cohort, including symptomatic patients with LV outflow obstruction who were treated conservatively. Notably, however, HCM-related mortality remained low in absolute terms for the study cohort (1.3%/y), despite differences in management strategies over the long period of observation, with event rates less than currently reported for other chronic cardiac and noncardiac diseases, including HF due to ischemic or primary dilated cardiomyopathy.^19^^,^^20^ Furthermore, arrhythmic SCD occurred in only 8% (16/202) of patients, generally at younger ages than HF deaths, and without increased incidence over time, in part due to the introduction of prophylactic ICDs into the cohort.^1^^,^^2^^,^^15^^,^^21^

Thus, while our data are largely consistent with the established paradigm of HCM as a chronic disease with relatively low lifetime risk of lethal events,^1^^,^^2^ the present study adds a measure of novelty by its much longer follow-up duration. Prior reports with follow-up <10 years from diagnosis potentially underestimate long-term HCM-related complications and mortality, which in the present cohort more than doubled after the first decade. This temporal trend finds support in the structural cardiac remodeling we observed over the years in some patients, including progressive left atrial dilatation and decrease in ejection fraction associated with the end-stage.^19^ Notably, a specific analysis taking into account cardiac (presence of nonsustained ventricular tachycardia, syncope or coronary artery disease) and noncardiac factors (cancer death, chronic obstructive pulmonary disease, and chronic kidney disease), possibly influencing all-cause, HCM, HF/stroke related, and SCD mortality were performed using a Fine-Gray regression model (Table 2). Such competing variables did not influence HCM-related mortality, which was in fact driven by disease-related factors such as HF. This is supported by the finding that the only independent predictor of HCM-related mortality was confirmed to be the presence of a LA dilation >45 mm (Table 2).^22^^,^^23^

While a subset of patients in this study benefited substantially from life-saving treatment options (ICDs and septal reduction), we also acknowledge a limitation in that our mortality data do not fully reflect the potential of those management options available to HCM patients today. Therefore, we have considered those cases in which deaths were potentially preventable based on a contemporary approach. These include patients with one or more established SCD risk factors who died suddenly without protection from ICDs; fatal cardioembolic strokes in patients with AF not receiving prophylactic oral anticoagulation; patients aged <65 years who were potentially eligible for heart transplantation and were not transplanted; and patients in NYHA functional classes III-IV and LV outflow gradient ≥50 mm Hg eligible for myectomy surgery but managed pharmacologically. Notably, in this regard, one-third of the relatively young patients in our cohort with obstructive HCM amenable to septal reduction therapies were treated conservatively in the early years (usually medically with negative inotropic drugs) without ready access to myectomy, likely explaining our morbidity and mortality rates reported in the second/third decades of follow-up.

These data also support recognition that contemporary management options can substantially impact mortality directly attributable to HCM.^2^^,^^20^^,^^24^ Indeed, >40% of our patients would have benefited importantly by treatments not available in many cases >20 years ago.^20^ However, nevertheless, several real-world clinical challenges remain. While systematic implementation of strategies such as septal reduction therapy, oral anticoagulation in cardioembolic prevention, or the ICD for SCD prevention are now widely disseminated, treatment of advanced HF in nonobstructive HCM remains logistically challenging due to limited access to heart transplantation and other advanced HF treatments.^25^, ^26^, ^27^, ^28^

However, taken together, these considerations largely explain the difference between the HCM mortality rate of 1.3%/y reported here vs 0.5%/y characteristic of other HCM cohorts with access to all contemporary treatment strategies including low-risk/high-benefit surgical myectomy^1^^,^^2^ and primary prevention ICDs^17^ in a best care “real world” clinical practice model.^2^

### Study limitations

The aim of the present study was to determine the natural history and extended outcome of HCM patients identified very early in the experience with the disease. As a retrospective study spanning many decades and different treatment eras, certain current advanced treatment options for HCM, such as surgical myectomy, heart transplant and ICDs, were not widely available in Central Italy for portions of the extended follow-up period. However, this recognition reflects the reality of our ‘real-world assessment’ of HCM up to 50 years, and the importance of such treatments for patients with this disease.

## Conclusions

In this unique consecutive HCM cohort followed continuously for up to 50 years, including periods of time before certain current effective therapeutic options became widely available, we found that HCM was not a uniformly progressive disorder. While the risk of HF increased over time, the risk of arrhythmic sudden death remained constant. Despite the overlapping management eras spanning over 5 decades, HCM has a relatively low annual mortality risks, albeit higher than what is currently achievable.

## Funding support and author disclosures

Dr Olivotto was supported by the European Union’s Horizon 2020 Research and Innovation Programme under Grant Agreement no. 777204: “SILICOFCM - In Silico trials for drug tracing the effects of sarcomeric protein mutations leading to familial cardiomyopathy”; has received grants from 10.13039/100002491Bristol Myers Squibb, 10.13039/100014941Cytokinetics, 10.13039/100015362Amicus, 10.13039/100004329Genzyme, 10.13039/100007343Shire, 10.13039/100004326Bayer, 10.13039/100008497Boston Scientific, Menarini International; fees (honoraria or consulting) from Bristol Myers Squibb, Cytokinetics, Amicus, Genzyme, Shire, and Boston Scientific; and served or currently serving as PI on EXPLORER-HCM, MAVA-LTE, REDWOOD-HCM, REDWOOD-OLE, and SEQUOIA-HCM trial. Dr M. S. Maron is a consultant for Cytokinetics and Steering Committee Chair for REDWOOD-HCM phase 2 trial; consultant for Imbria Pharmaceuticals; and is a consultant and has a research grant from 10.13039/100007723Takeda Pharmaceuticals. All other authors have reported that they have no relationships relevant to the contents of this paper to disclose.

PERSPECTIVES **COMPETENCY IN PATIENT CARE:** Current understanding of the clinical course and long-term outcome of patients with HCM has been extrapolated from cohorts with relatively short follow-up, usually <10 years. In this unique HCM cohort followed for up to 50 years, often before contemporary therapies became widely implemented for HCM, annual overall HCM-related mortality was 1.3%/y, increasing from 0.7% during the first decade to 1.8% in the second/third decade, mainly driven by increase in HF-/stroke-related events (from 0.6% to 1.3%). However, sudden death mortality rate was similar during the 2 periods (0.1% vs 0.44%).

**TRANSLATIONAL OUTLOOK:** These data support the recognition that contemporary management options can substantially impact mortality directly attributable to HCM. Indeed, >40% of our patients would have benefited importantly by treatment options not fully available >20 years ago.

## References

[1] Maron B.J. (2018). Clinical course and management of hypertrophic cardiomyopathy. N Engl J Med.

[2] Maron B.J., Rowin E.J., Casey S.A., Maron M.S. (2016). How hypertrophic cardiomyopathy became a contemporary treatable genetic disease with low mortality: shaped by 50 years of clinical research and practice. JAMA Cardiol.

[3] Maron B.J., Olivotto I., Spirito P. (2000). Epidemiology of hypertrophic cardiomyopathy-related death: revisited in a large non-referral-based patient population. Circulation.

[4] Maron B.J., Rowin E.J., Casey S.A. (2016). Hypertrophic cardiomyopathy in children, adolescents and young adults associated with low cardiovascular mortality with contemporary management strategies. Circulation.

[5] Maron M.S., Olivotto I., Zenovich A.G. (2006). Hypertrophic cardiomyopathy is predominantly a disease of left ventricular outflow tract obstruction. Circulation.

[6] Maron M.S., Rowin E.J., Olivotto I. (2016). Contemporary natural history and management of nonobstructive hypertrophic cardiomyopathy. J Am Coll Cardiol.

[7] Spirito P., Bellone P., Harris K.M., Bernabo P., Bruzzi P., Maron B.J. (2000). Magnitude of left ventricular hypertrophy and risk of sudden death in hypertrophic cardiomyopathy. N Engl J Med.

[8] Maron M.S., Olivotto I., Betocchi S. (2003). Effect of left ventricular outflow tract obstruction on clinical outcome in hypertrophic cardiomyopathy. N Engl J Med.

[9] Desai M.Y., Bhonsale A., Patel P. (2014). Exercise echocardiography in asymptomatic HCM: exercise capacity, and not LV outflow tract gradient predicts long-term outcomes. J Am Coll Cardiol Img.

[10] Espinola-Zavaleta N., Vega A., Basto D.M., Alcantar-Fernández A.C., Guarner Lans V., Soto M.E. (2015). Survival and clinical behavior of hypertrophic cardiomyopathy in a Latin American cohort in contrast to cohorts from the developed world. J Cardiovasc Ultrason.

[11] Maurizi N., Passantino S., Spaziani G. (2018). Long-term outcomes of pediatric-onset hypertrophic cardiomyopathy and age-specific risk factors for lethal arrhythmic events. JAMA Cardiol.

[12] Sugiura K., Kubo T., Ochi Y. (2022). Very long-term prognosis in patients with hypertrophic cardiomyopathy: a longitudinal study with a period of 20 years. ESC Heart Fail.

[13] Ho C.Y., Day S.M., Ashley E.A. (2018). Genotype and lifetime burden of disease in hypertrophic cardiomyopathy: insights from the Sarcomeric Human Cardiomyopathy Registry (SHaRe). Circulation.

[14] Cecchi F., Olivotto I., Montereggi A., Santoro G., Dolara A., Maron B.J. (1995). Hypertrophic cardiomyopathy in Tuscany: clinical course and outcome in an unselected regional population. J Am Coll Cardiol.

[15] Braunwald E., Lambrew E., Rockoff D. (1964). Idiopathic hypertrophic subaortic stenosis. A description of the disease based upon an analysis of 64 patients. Circulation.

[16] Maron B.J., Shen W.K., Link M.S. (2000). Efficacy of implantable cardioverter-defibrillators for the prevention of sudden death in patients with hypertrophic cardiomyopathy. N Engl J Med.

[17] Maron M.S., Rowin E.J., Wessler B.S. (2019). Enhanced American College of Cardiology/American Heart Association strategy for prevention of sudden cardiac death in high-risk patients with hypertrophic cardiomyopathy. JAMA Cardiol.

[18] Fine J.P., Gray R.J. (2012). A proportional hazards model for the subdistribution of a competing risk. J Am Stat Assoc.

[19] Castelli G., Fornaro A., Ciaccheri M. (2013). Improving survival rates of patients with idiopathic dilated cardiomyopathy in Tuscany over 3 decades: impact of evidence-based management. Circ Heart Fail.

[20] Maron B.J., Maron M.S., Rowin E.J. (2017). Perspectives on the overall risks of living with hypertrophic cardiomyopathy. Circulation.

[21] Harris K.M., Spirito P., Maron M.S. (2006). Prevalence, clinical profile, and significance of left ventricular remodeling in the end-stage phase of hypertrophic cardiomyopathy. Circulation.

[22] Uretsky S., Bangash A., Rowin E. (2021). Determinants of LV dilatation in patients with hypertrophic cardiomyopathy and preserved systolic function: a CMR study. J Am Coll Cardiol Img.

[23] Nistri S., Olivotto I., Betocchi S. (2006). Prognostic significance of left atrial size in patients with hypertrophic cardiomyopathy (from the Italian Registry for Hypertrophic Cardiomyopathy). Am J Cardiol.

[24] Maron B.J., Rowin E.J., Maron M.S. (2021). Evolution of risk stratification and sudden death prevention in hypertrophic cardiomyopathy: twenty years with the implantable cardioverter-defibrillator. Heart Rhythm.

[25] Rowin E.J., Maron B.J., Romashko M. (2019). Impact of effective management strategies on patients with the most extreme phenotypic expression of hypertrophic cardiomyopathy. Am J Cardiol.

[26] Biagini E., Spirito P., Leone O. (2008). Heart transplantation in hypertrophic cardiomyopathy. Am J Cardiol.

[27] Rowin E.J., Maron B.J., Abt P. (2018). Impact of advanced therapies for improving survival to heart transplant in patients with hypertrophic cardiomyopathy. Am J Cardiol.

[28] Rowin E.J., Maron B.J., Carrick R.T. (2020). Outcomes in patients with hypertrophic cardiomyopathy and left ventricular systolic dysfunction. J Am Coll Cardiol.

