# Supplementary material for: Lifetime Clinical Course of Hypertrophic Cardiomyopathy: Outcome of the Historical Florence Cohort Over 5 Decades
Source: JACC Adv. 2023 May 24;2(4):100337. doi: 10.1016/j.jacadv.2023.100337 (PMC11198069; doi:10.1016/j.jacadv.2023.100337)

Supplemental Table 1. Clinical characteristics of the 202 HCM patients at baseline and at last available contact during follow-up.

	Variable	Enrollment (n=202)	At Last available contact (n=189)
**Medical History**			
	Age (years)	41± 17	65 ± 14
	Aged more than 70 years (n)	7 (%)	108 (74%)
	Male sex (n)	140 (69%)	133 (65%)
	NYHA I - II	183 (90%)	141 (75%)
	NYHA III -IV	19 (10%)	52 (27%)
	LVOT Gradient > 30mmHg (n)	40 (20%)	54 (29%)
	LVOT Gradient ≥ 50mmHg (n)	23 (11%)	28 (15%)
	Syncope (n)	8 (4%)	45 (24%)
	Non sustained VT (n)	22 (11%)	68 (36%)*
	Invasive septal reduction (n)	0	14 (7%)
	Atrial Fibrillation (n)	21 (10%)	83 (44%)*
	ICD implantation (n)	0	32 (17%)*
	Sarcomeric positive (n)	0	61/125(48%)
**Drugs**			
	Beta – Blockers (n)	18 (9%)	146 (85%)*
	Verapamil (n)	4 (2%)	13 (7%)
	Dysopiramide (n)	0	27 (14%)
	Amiodarone (n)	8 (4%)	74 (40%)
	ACE Inhibitor (n)	1 (0.1%)	18 (10%)
	Anti-coagulation (n)	17 (8%)	91 (48%)*
**Echocardiography**			
	LA diameter (mm)	40 ± 6	49 ± 9 *
	EF (%)	65 ± 9	54 ± 12 *
	Maximal LV thickness (mm)	23 ± 5	19 ± 4 *
	LV thickness ≥ 30 mm (n)	24 (11%)	19 (10%)
	Restrictive diastolic pattern (n)	12 (6%)	44 (23%)*

*Abbreviations: LVOT = left ventricular outflow tract; VT = ventricular tachycardia on ambulatory ECG; ICD = implantable cardioverter defibrillator; LA = left atrium; LV = left ventricle; EF= ejection fraction; LV = left ventricle; NYHA = New York Heart Association. Symbols: * = p < 0.05-0.001.*

Supplemental Figure 1. Age at death of the cohort stratified per decade

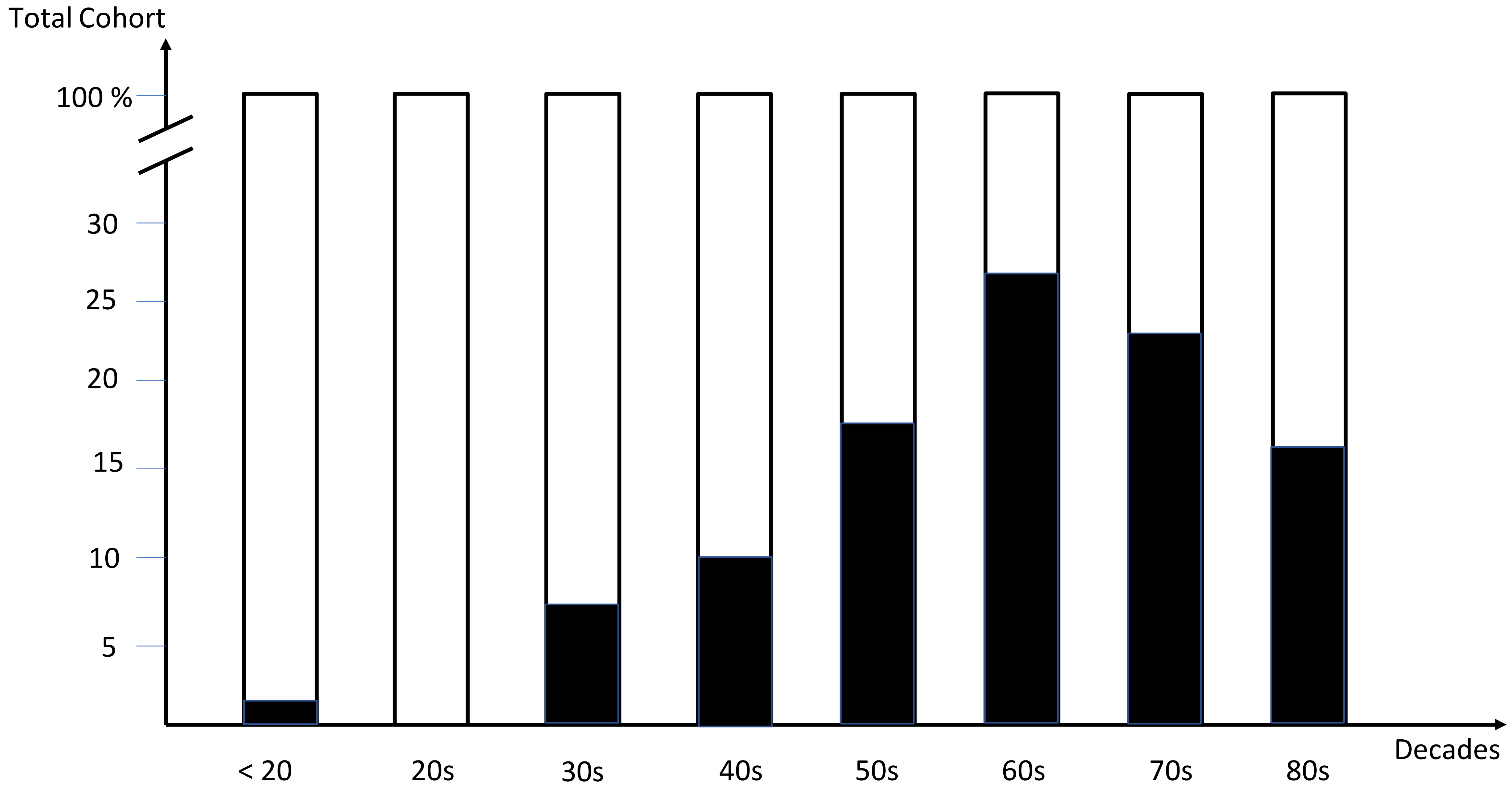

Supplemental Figure 2. Age at death of the HCM cohort and age-matched italianpopulation from the same geographical region.

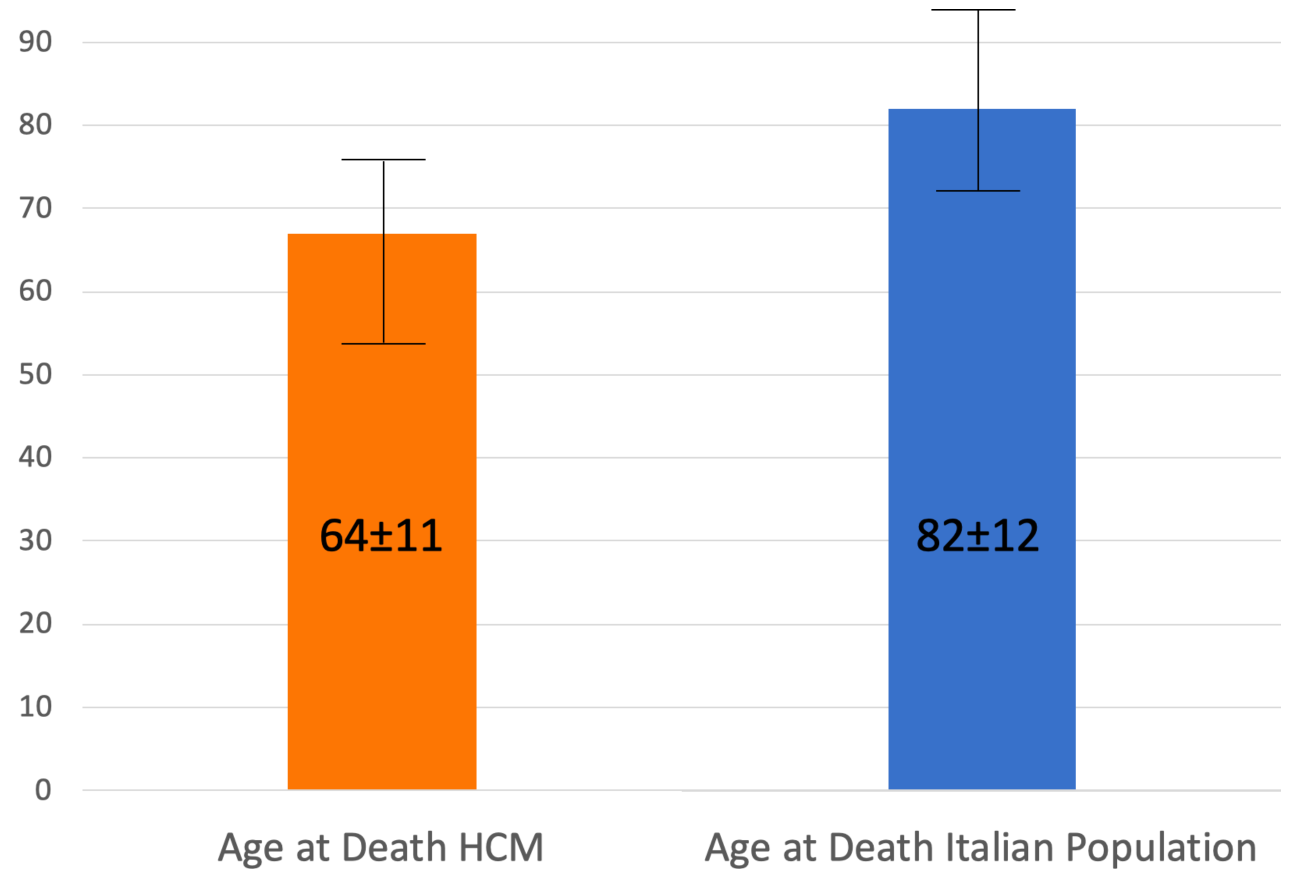

Supplement: Supplemental Figures 1, 2 and Table 1 [file mmc1.pdf]
